# Beta alanine supplementation effects on metabolic contribution and swimming performance

**DOI:** 10.1186/s12970-020-00365-6

**Published:** 2020-07-25

**Authors:** Matheus Silva Norberto, Ricardo Augusto Barbieri, Danilo Rodrigues Bertucci, Ronaldo Bucken Gobbi, Eduardo Zapaterra Campos, Alessandrou Moura Zagatto, Ellen Cristini De Freitas, Marcelo Papoti

**Affiliations:** 1 University of São Paulo, Medicine University of Ribeirão Preto (FMRP-USP), Ribeirão Preto, São Paulo Brazil; 2 University of São Paulo, School of Physical Education and sport of Ribeirão Preto (EEFERP-USP), Ribeirão Preto, São Paulo Brazil; 3 Estácio University, Ribeirão Preto, São Paulo Brazil; 4 Department of Physical Education, State São Paulo University, (UNESP), Rio Claro, São Paulo Brazil; 5 Department of Physical Education, Federal University of Pernambuco, (UFPE), Recife, Pernambuco Brazil; 6 Department of Physical Education, State São Paulo University, (UNESP), Bauru, São Paulo Brazil

**Keywords:** Beta-alanine, Swimming, Anaerobic energy release, 30-s all-out tethered, Accumulated oxygen deficit, Sports science

## Abstract

**Background:**

Investigations of β-alanine supplementation shows effects on metabolic (aerobic and anaerobic) participation and performance on swimming by a possible blood acidosis buffering. Considering this background, the objective of the present study was to analyze the effects of β-alanine supplementation on metabolic contribution and performance during 400-m swim.

**Methods:**

Thirteen competitive swimmers underwent a 6-week, double-blind placebo-controlled study, ingesting 4.8 g.day^− 1^ of β-alanine or placebo. Before and after the supplementation period, the total anaerobic contribution (TAn) and 30-s all-out tethered swimming effort (30TS) were assessed. Anaerobic alactic (AnAl) and lactic energy (AnLa) was assumed as the fast component of excess post-exercise oxygen consumption and net blood lactate accumulation during exercise (∆[La^−^]), respectively. Aerobic contribution (Aer) was determined by the difference between total energy demand and TAn. In addition to conventional statistical analysis (Repeated measures ANOVA; *p* > 0.05), a Bayesian repeated measures ANOVA was used to evidence the effect probability (BF_incl_).

**Results:**

No differences and effects were found between groups, indicating no supplementation effects. Repeated measures ANOVA, with confirmation of effect, was indicate reduce in ∆Lactate (p: 0.001; BF_incl_: 25.02); absolute AnLa (p: 0.002; BF_incl_: 12.61), fatigue index (*p* > 0.001; BF_incl_: 63.25) and total anaerobic participation (p: 0.008; BF_incl_: 4.89).

**Conclusions:**

Thus, the results demonstrated that all changes presented were evidenced as a result of exposure to the training period and β-alanine supplementation doesn’t affect metabolic contribution and performance during 400-m freestyle.

## Key points of the paper

- Training period alters energy provision, neuromuscular and metabolic parameters
- Beta-alanine supplementation demonstrated no effects on energy provision to 400m freestyle swimming performance.
- Beta-alanine supplementation doesn’t alter force parameters on tethered swimming test
- Beta-alanine supplementation doesn’t alter performance on 400m freestyle swimming

## Introduction

Effort in 400-m swimming is highly intense (i.e. 83.2% aerobic, 10.2% anaerobic lactic, 5.8% anaerobic alactic) [1]. Despite the aerobic predominance, a high demand of glycolytic energy is required and athletes must tolerate high blood lactate concentrations ([La^−^]) (i.e., 8.5 ± 3.2 mmol/L [2]) for approximately 4 min, estimated time at which maximum hydrogen ions (H^+^) accumulation and low intramuscular pH are reported [3]. In this sense, nutritional supplementation seems to be a good strategy to delay fatigue [4, 5]. Several substances such as bicarbonate, phosphates, caffeine, proteins and amino acids have been used in sports that present high H^+^ concentration, inducing muscle acidosis and, consequently, fatigue [5–7].

Beta-alanine (β-alanine) supplementation has been widely used as an ergogenic strategy by competitive athletes from different sports [5, 6, 8] and seems capable to increase (~ 65% after 4 weeks) intramuscular carnosine content (β-alanyl-L-histidine), a cytoplasmic dipeptide found in human skeletal muscle [9, 10]. Since the buffering capacity pKa is ~ 6,1 [11] and carnosine is ~ 6,83 [12], it is assumed that carnosine is a physical-chemical buffer acting inside skeletal muscle where acidosis can be buffered from baseline status (pH ~ 7.1) to fatigue (pH ~ 6.5) during high-intensity exercises [5, 13, 14]. Some studies evidenced that 4 to 6 g∙d^− 1^ of β-alanine supplementation is sufficient to increase muscle carnosine content as from the fourth week [10, 15].

During supra maximal exercises, β-alanine supplementation may be capable of promoting changes in metabolic contribution and an improvement on power production rate, possibly by an increase in calcium (Ca^++^) released by the sarcoplasmic reticulum [16].

Among the studies that reported improvement on performance [4, 6, 16, 17], a study showed slight enhancement of 100-m and 200-m effort in swimmers after 4 weeks of β-alanine supplementation [17]. However, Chung et al. (2012) showed an unclear effect on performance fir the same distance after 10 weeks of supplementation. Therefore, these results in 200-m performance after β-alanine supplementation may have occurred mainly because of the distance (longer) and high intensity, considering that acidosis plays an important role on fatigue, and suggest that buffering capacity provided by β-alanine supplementation can be more efficient in longer efforts [4, 7, 17, 18].

Recent evidences indicate that β-alanine supplementation may alter metabolism, particularly the glycolytic pathway during supra-maximal stress [6, 18–20] and may [18, 21], or not [20], result in performance improvement. Although β-alanine performance improvements have been reported, this scenario is only found in specific experimental designs and laboratory conditions with low applicability in daily training [22].

Since β-alanine induces ergogenic effect in highly-trained athletes during sports whose performance may influenced and limited by acidosis, this study hypothesized that β-alanine, in theory, is capable of improving 400 m performance due to an increase in total anaerobic contribution (TAn) and / or improvement of the swimmers’ propulsive force maintenance.

Considering this, investigations of β-alanine supplementation effects on metabolic (aerobic and anaerobic) participation and performance in a “real life” situation are needed, especially for coach decision-making regarding the use of such nutritional strategies to improve swimmers’ performance. Therefore, the objective of the present investigation was to verify the effects of a 6-week β-alanine supplementation on metabolic contribution, force and swimming performance parameters.

## Methods

### Participants

Using software G*Power (version 3.1.1.9 – Universitat Kiel, Germany), it was possible to identify that 13 participants were necessary for the present study to obtain a significant statistical power (sample power of 0.80 and alpha of 0.05). Sixteen competitive swimmers, with at least 4 years of experience in national level competitions, volunteered to take part in the present study. Despite the difference in swim specialty, the 400-m performance of the participating swimmers represents 76.5% of the current 25-m pool world record for men and 77.1% for women. Only the data regarding the athletes who completed all supplementation, training period and performed all tests were analyzed. Based on this, three athletes were withdrawn from the study, resulting in thirteen swimmers, eight males (20.25 ± 1.98 years; 78.16 ± 7.03 kg and $\dot{\mathrm{V}}$ O_2_peak of 61.20 ± 6.02 ml.kg^− 1^.min^− 1^) and five females (20.0 ± 2.92 years, 59.96 ± 9.50 kg; and $\dot{\mathrm{V}}$ O_2_peak of 49.58 ± 7.83 ml.kg^− 1^.min^− 1^).

All participants and/or their legal guardians were informed about the risks and benefits of participating in the present study, and both provided a written informed consent authorizing the athletes to undertake the experiments. Participants were recruited provided they were not taking any chronic or daily doses of nutritional supplements or anti-inflammatory medication. In addition, the athletes were instructed to avoid drinking caffeine, alcohol, and energy drinks for at least 12 h before each measurement.

### Protocol

A 6-week double blind, placebo-controlled study design was conducted. During the supplementation period both groups underwent the same training, so both groups may have performance improvement, however, it is expected that the addition of β-alanine promotes extra performance improvement. Swimmers performed a 400-m maximal freestyle bout and a 30-s all-out tethered swimming effort (30TS) with a 48-h interval between each effort, before and after the supplementation period.

Participants were randomly separated (by an draw) into placebo (PLA) and β-alanine groups. The substances were packed in gastro-resistant capsules (hydroxypropylmethylcellulose, DrCapsTM, Capsugel, Puebla, PUE, Mexico), identical in appearance, size and weight, containing 800 mg of β-alanine (99.9% pure β-alanine, CarnoSyn™, NAI, San Marcos, CA, USA) or starch for PLA. Athletes were instructed to take the capsules (6 capsules per day × 800 mg = 4.8 g.day^− 1^) throughout the day, with a minimum of 2 h between each capsule to avoid paresthesia.

All procedures were conducted in a 25-m swimming pool with water temperature of 26 ± 1 °C. Before all trials, a standardized warm-up was performed consisting of approximately 1000-m, with exercises to develop technique, swimming sensibility and high intensity stimulus, elaborated by the squad head coach. Before the 400-m effort, baseline oxygen consumption was assessed during 1 min at rest and immediately after the 400-m effort for 5 min. Performance (time and velocity) was analyzed through a motion-analysis on software Kinovea™ (version 0.8.15).

Oxygen measurements: Expired gases were assessed breath-by-breath using a Quark CPET gas analyzer (Cosmed®, Rome, Italy) using backward extrapolation technique [1]. The gas analyzer was calibrated pre-exercise in accordance with the manufacturer’s instructions. After the 400-m performance, athletes were instructed to breathe immediately into the facemask (Hans Rudolph, Kansas City, MO, USA), connected to the gas analyzer.

After removal of outliers (exclude discrepant breaths), breath-by-breath data were interpolated (OriginPro 8.0, OriginLab Corporation®, Microcal, Massachusetts, USA) to enhance underlying responsive characteristics. V̇O_2_ values were log-transformed and plotted against time, which was linearly adjusted. Thus, the y-intercept was considered as V̇O_2_ at the end of exercise [23–25], and assumed as the first point of recovery.

Anaerobic energy estimative: To calculate fast component of excess post-exercise oxygen consumption (EPOC_FAST_) values, V̇O_2_ accessed during 5 min of recovery was adjusted by a bi-exponential decay fit [24, 26, 27]. Anaerobic alactic contribution (AnAl), represented by EPOC_FAST_, was assumed as the product of the amplitude and time constant of the first exponential decay [27].

Immediately before, 3, 5 and 7 min after the 400-m performance, blood samples (25 μl) were collected from the earlobe to determine [La^−^] at rest ([La^−^]_rest_) and peak ([La^−^]_PEAK_). The amount of blood lactate accumulation was the difference between [La^−^] at peak and baseline values (∆[La^−^]). [La^−^] was determined using a blood analyzer YSI-2300 (Yellow Springs Instruments®, Ohio, USA).

Anaerobic lactic contribution (AnLa) was determined by ∆[La^−^], considering a metabolic equivalent of 3 mL∙O2∙^− 1^ kg^− 1^ for each unit of lactate accumulated during the maximal effort [28]. Aerobic contribution (Aer) was determined by the difference between total energy demand and TAn [27, 29].

30-s all-out tethered swimming test: All swimmers have large experience in 30TS, being applied during training and evaluations throughout the competitive season. Peak force (PF), mean force (MF), fatigue index (FI(%) = ((PF – minimum force)*100)/PF) and impulse (IM = integral of the relation between force and time during the intervals that represent each stroke) were determined in 30TS. A loadcell (CSR-100 kg, MK Controle® São Paulo, Brazil), previously calibrated with six known weights, was connected a belt to the swimmer using 6-m inextensible nylon cord. Values obtained by 30TS were sent to a data acquisition device (NI-USB-6008, National Instrument Corporation; Austin, Texas, USA), then processed by LabVIEW 8 (National Instruments Corporation; Austin, Texas, USA) at 400 Hz.

This procedure started with 5 s low intensity swim to fully extend the cable, after a sonorous signal the swimmers maintain maximal effort in the next 30 s until another signal determined the end of the test. The first maximum cycle was rejected to avoid inertial effect extending the cable. Athletes were strongly encouraged to maintain maximum effort during the test and had no feedback in remaining time and force production.

Training characteristics: The swimming squad participating in the present study performed the same training program developed by the coaching staff and trained in the pool six times a week. Prior to to the supplementation period, athletes underwent a base training phase, in which the main objective was to develop aerobic metabolism to support the specific training phase. Base training was characterized by high volume (7000 m to 10,000 m a day) and low intensity (below anaerobic threshold), high intensity bouts were rare during this phase. After baseline measurements, athletes began the supplementation period concomitantly to a specific training phase, which consisted of a 1000 m to 1500 m warm-up and focus on swimming technique, followed by a main part and finishing the session with an approximately 1000 m cooldown. Most part of the session consisted in different training stimuli, such as high volume (4000 m) and low intensity (below anaerobic threshold) (i.e. Z1); a slight decrease in volume compared to Z1 (3000m) at anaerobic threshold intensity (i.e. Z2); low volume (1500 m) at high intensity (above anaerobic threshold intensity) (i.e. Z3), or very low volume (600 m) at maximum intensity (i.e. anaerobic training). Each week comprised a different set of stimuli according to the specific week goal. During this phase, swimmers performed resistance training 4 times a week in the strength and conditioning room. Both groups performed same training program and nearly 70% of the training sessions were performed at Z1, while the other 30% represent sessions at higher intensities and lower volume (Z3 anaerobic training), characterizing a polarized training distribution [30].

### Statistical analysis

Statistical analyses were performed using JASP (Amsterdam, Netherland) software version 0.9.1. Data are presented as mean ± SD. Repeated measures between-subjects ANOVA for each dependent variable was performed using Time (Pre/Post) vs Supplementation (β-alanine/PLA). Statistical significance was fixed at *p* < 0.05. In addition to compare the difference between the pre and post-training variables between the BA and PLA groups a Bayesian repeated measures ANOVA approach was used to provide probabilistic statements of the supplementation effect was used to compare difference change between BA and PLA variables. Evidence for the alternative hypothesis (H1) was set as BF_incl_ > 3 and evidence for null hypothesis (H_0_) was set as BF_incl_ < 1/3. BF_incl_ was reported to indicate the strength of the evidence for each analysis. The BF_incl_ was interpreted using the following evidence categories: 1 < BF_incl_ < 3 = *anecdotal* evidence for H_1_; BF_incl_ ≥ 3 = *moderate*; BF_incl_ ≥ 10 = *strong*; BF_incl_ ≥ 30 = *very strong*; BF_incl_ ≥ 100 = *extreme* [31].

Additionally, the standard error of measurement (SWC) analysis was used in order to establish the percentage of evaluated that showed difference in the analyzed parameters. Because the typical error (TE) was higher than the SWC, to ensure the validity of the calculation, we used TE as an “alteration index”. The TE was based on an existing database in our laboratory based on test-retest of the analyzed parameters.

## Results

Three participants in β-alanine group reported paresthesia. Repeated measures between-subjects ANOVA showed no significant differences for the variables. Differences after the supplementation and training period was found for β-alanine and PLA groups on absolute MAOD (p: 0.008), absolute anerobic lactic contribution (p: 0.002), percentile of anaerobic lactic contribution (p: 0.022), percentile of anaerobic alactic contribution (p: 0.032), ∆Lactate (p: 0.001) and VO_2_max (p: 0.045). Analysis of difference in energy provision and metabolic parameters are shown in Table 1.

**Table 1:** Analyses of the difference in energy provision and metabolic parameters before and after the supplementation period for both groups (mean ± standard deviation) and the respective significance value

Parameters	Suppl.	Moment		Significance value (*p*)		
		Pre	Post	Moment	Supplementation	Interaction
Energy provision parameters						
MAOD (L)	BA	3.84 ± 1.4	2.94 ± 1.2	0.008*	0.639	0.160
	PLA	3.86 ± 1.2	2.94 ± 0.9			
Aer. contribution (L)	BA	14.3 ± 4	13.3 ± 3.1	0.128	0.141	0.921
	PLA	13.7 ± 3.8	12.6 ± 2.9			
Ana. lactic contribution (L)	BA	2.27 ± 0.9	1.5 ± 0.6	0.002*	0.685	0.179
	PLA	2.26 ± 0.9	1.9 ± 0.8			
Ana. alactic contribution (L)	BA	1.32 ± 0.4	1.1 ± 0.6	0.056	0.415	0.342
	PLA	1.35 ± 0.4	1.4 ± 0.8			
Aer. contribution (%)	BA	82.1 ± 6.3	83.7 ± 6.3	0.960	0.453	0.276
	PLA	81.3 ± 4.1	79.4 ± 8.5			
Ana. lactic contribution (%)	BA	12.9 ± 4.7	9.5 ± 3.1	0.022*	0.520	0.356
	PLA	13.4 ± 3.8	11.8 ± 4.2			
Ana. alactic contribution (%)	BA	4.9 ± 2.3	6.6 ± 3.2	0.032*	0.413	0.414
	PLA	5.2 ± 2.2	8.7 ± 4.3			
Metabolic parameters						
∆Lactate (mM)	BA	11.6 ± 3.6	8 ± 2.4	0.001*	0.762	0.166
	PLA	11.2 ± 3.1	9.46 ± 3.3			
Total energetic cost (L)	BA	19.6 ± 4.4	18.1 ± 3.4	0.062	0.945	0.623
	PLA	19.2 ± 4.4	18.26 ± 3.4			
VO_2_max (ml.kg.min^−1^)	BA	58.8 ± 9.3	54.1 ± 6.0	0.045*	0.942	0.650
	PLA	54.2 ± 8.0	51.7 ± 4.8			

Variables values expressed in means ± standard deviation. *Suppl.* supplementation; *p < 0.05

No difference was found for performance after the supplementation and training period. Significant differences were also found after the supplementation and training period for percentile of fatigue index (*p* < 0,001). Analysis of difference in performance and neuromuscular parameters.

Using a Bayesian approach, the effect analysis shown no effects of supplementation or interaction in performance, neuromuscular, metabolic and energy provision parameters. The training period was shown reduce effect on absolute MAOD, absolute anaerobic lactic contribution, ∆Lactate and percentile of fatigue index. The proportions of the real effect analyzed was shown on Table 3.

Table 4 shows in details the incidence of participants who were exposed to a change outside the normative limit (typical measurement error). That is, the percentage of swimmers who obtained a change that may indicates a change provided by the temporal effect (exposure to training and / or supplementation).

## Discussion

The present study investigated β-alanine ergogenic potential applied on a “real life” and training/competitive environment in swimming [4, 7, 17] and the energy provision during 400-m with this supplementation. The main findings were that β-alanine supplementation doesn’t result in metabolic contribution changes and it was ineffective in improving swimming performance.

No potential effect of β-alanine supplementation on force parameters or performance were observed. It is important to emphasize that few studies analyzed only β-alanine on competitive swimmers [4, 7]. A study involving water polo evidenced that although β-alanine results in increased force associated with peak oxygen uptake (V̇O_2PEAK_), this improvement does not result in improved swimming performance or metabolic changes [20]. Evidencing the association between VO_2_ and increased strength in water sports [20], it is important to note that, in the present study, the training period coincided with a reduction in VO_2_max of both groups of swimmers (Table 1). Although the “classic” approach indicates a difference between the pre and post moments (Table 2; p: 0.045), a possible effect was not detected (Table 3; BF_incl_: 1.563), indicating that in this study this interaction may not exist.

**Table 2:** Analyses of the difference in neuromuscular parameters before and after the supplementation period for both groups (mean ± standard deviation) and the respective significance value

Parameters	Suppl.	Moment		Significance value (*p*)		
		Pre	Post	Moment	Supplementation	Interaction
Performance (s/400m)	BA	288.9 ± 18.6	289.2 ± 21.0	0.913	0.799	0.892
	PLA	292.2 ± 22.6	292.1 ± 24.2			
Peak force (N)	BA	236.0 ± 37.6	210.1 ± 44.6	0.456	0.520	0.688
	PLA	238.2 ± 66.7	230.3 ± 47.8			
Mean force (N)	BA	95.7 ± 16.6	103.3 ± 23.7	0.269	0.936	0.649
	PLA	90.7 ± 35.2	106.3 ± 33.1			
Impulse (N·s)	BA	2810.8 ± 477	3016.5 ± 707.6	0.246	0.952	0.620
	PLA	2642.7 ± 1024.2	3141.6 ± 986.8			
Fatigue index (%)	BA	57.7 ± 5.7	49.5 ± 7.3	< 0.001**	0.419	0.799
	PLA	61.4 ± 12.7	54.1 ± 10.5			

Variables values expressed in means ± standard deviation. *Suppl.* supplementation; ** *p* < 0,001

**Table 3:** Difference in percentile of performance, energy provision, metabolic and neuromuscular parameters after the supplementation period and the respective bayesian analysis of effect

Parameters	Difference (%)		Effect analysis by Bayes factor (BF_incl_)		
	BA	PLA	Moment	Supplementation	Interaction
Energy provision and metabolic parameters					
MAOD (L)	−23.3	−8.3	4.895^a^	0.788	1.217
Aer. contribution (L)	−7.1	−8.4	0.742	0.517	0.364
Ana. lactic contribution (L)	−32.7	−15.3	12.615^b^	0.764	1.227
Ana. alactic contribution (L)	23.1	63.2	1.367	0.507	0.570
Aer. contribution (%)	2.1	−2.3	0.301	0.507	0.284
Ana. lactic contribution (%)	−26.3	−11.9	2.963	0.596	0.735
Ana. alactic contribution (%)	34.2	66.8	2.724	0.552	0.662
∆Lactate (mM)	−31.5	−15.8	25.020^b^	0.692	1.221
Total energetic cost (L)	−7.9	−4.9	1.259	0.570	0.492
VO_2_max (ml.kg.min^−1^)	− 7.8	−5.2	1.563	0.641	0.564
Performance and neuromuscular parameters					
Performance	0.11	−0.01	0.300	0.654	0.213
Peak force (N)	−10.9	−3.2	0.406	0.368	0.230
Mean force (N)	7.9	17.2	0.512	0.363	0.242
Impulse (N·s)	7.3	18.8	0.598	0.341	0.244
Fatigue index (%)	−14.1	−11.9	63.255^c^	0.716	0.782

Effect analysis was made by BF_incl_. *Suppl.* supplementation, *BA* Beta-alanine, *PLA* Placebo; ^a^moderate evidence; ^b^strong evidence; ^c^very strong evidence

Among the main findings of the present study, a likely reduction in ∆[La^−^] was found (Table 1), corroborating with some studies in different situations [6, 18]. However, this study showed very strong evidence that the reduction in ∆[La^−^] was a training effect (Table 3). Although most of the subjects who presented changes in this parameter were in the group that supplemented with β-alanine (Table 4), there was no effect by supplementation. Despite the reduction in ∆[La^−^] being a possible evidence of improved buffering, this change was not able to change 400 m performance. Based on the significant increase found for the alactic anaerobic contribution (Table 1), it is possible that, although a probabilistic effect was not evidenced (Table 3), this situation in addition to the reduction in ∆[La^−^] contemplates an improvement in the tolerance of shorter efforts, explaining the improvement in the fatigue index performed in 30s of tethered swimming.

**Table 4:** Incidence of participants who exceeded the limit of the typical error indicating a change outside the normative standard for the parameter. The typical error is based on a database acquired in specific test-retest approaches for each of the parameters analyzed in the present study. The typical error is presented in the same unit as its respective parameter

Parameters	N	TE	β-alanine (%)			Placebo (%)		
			Increase	Maintain	Reduce	Increase	Maintain	Reduce
Delta [Lac-]	14	2.5	0	42.8	57.1	0	66.6	22.3
Performance (s/400m)	16	4.0	14.2	71.4	14.2	33.3	33.3	33.3
VO2 max	25	0.3	0	71.4	28.5	0	66.6	33.3
MAOD	14	0.6	0	42.8	57.4	0	83.3	16.6
GET (L)	9	2.2	0	71.4	28.5	0	66.6	33.3
Aer. Contribution (L)	9	2.2	0	71.4	28.5	0	66.6	33.3
Ana. Lactic Contribution (L)	14	0.5	0	42.8	57.4	0	66.6	33.3
Ana. Alactic Contribution (L)	14	0.5	28.5	71.4	0	33.3	66.6	0
Aer. Contribution (%)	9	3.3	0	57.1	42.8	0	50	50
Ana. Lactic Contribution (%)	9	2.3	0	57.1	42.8	0	50	50
Ana. Alactic Contribution (%)	9	2.0	42.8	57.1	0	50	50	0
Peak Force (N)	40	15.0	28.5	28.5	42.8	16.6	16.6	66.6
Mean Force (N)	40	6.0	28.5	0	71.4	50	33.3	16.6
Impulse (N·s)	40	180.6	57.1	14.2	28.5	66.6	16.6	16.6
Fatigue Index (%)	20	6.1	0	42.8	57.1	0	50	50

*N* number of subjects involved in the typical error calculation, *TE* Typical error for the measure

Another relevant finding is regarding the alteration of different anaerobic parameters (Table 1). The decrease in Δ [La^−^] for both groups, may have influenced the decrease of AnLa percentile and increase the AnAl percentile, since the calculation responsible for quantifying this parameter involves lactatemia kinetics [27, 28]. Considering that mathematical components to calculate AnAl and represent EPOC_FAST_, a decrease of this variable may represent a possible energy compensation from both aerobic and/or anaerobic alactic metabolism [27–29]. Also, important to highlight that, although there are no differences between the groups, the group that supplemented with β-alanine was more likely to shown individual changes in the metabolic parameters (Table 4).

Despite the decreases indicated by anaerobic parameters (Table 1) and the individual changes (Table 4), the present study did not find alterations on the aerobic component, suggesting a failure in the metabolic balance, since these systems should be compensated in order to represent the total expenditure discharged during such an effort [27, 29]. The method presents good reproducibility to quantify anaerobic component [27]; however, the strategy to estimate aerobic contribution may be inadequate [27, 29].

The method used to analyze energy contribution is useful to change paradigms related to metabolic contribution, identify better pacing strategies and estimate individual capacity to develop anaerobic performance [32]. Thus, although this method presents some limitations, it allows athletes to perform exactly as they would in training and competition. Therefore, it allows to estimate energy contribution with a greater ecological validity, since the athlete performs an effort with a metabolic cart and a snorkel attached, and V̇O_2PEAK_ values are underestimated due to the changes in swimming technique (without turns, mechanical variations, etc.).

The absence of significant changes in swimming performance (i.e. β-alanine and PLA groups), best evidenced in the percentage of individual differences (Table 4), can be attributed to the training phase in which the tests were performed. During this training period, the total volume of training was distributed in a way that ~ 70% of the training sessions were performed with swimming, considered by the swimmers of easy intensity (i.e., blood lactate < 2 mM), and ~ 30% in high intensity (blood lactate > 4 mM). Hence, the supplementation period was carried out during specific training periods. No measurements were conducted after taper.

β-alanine supplementation has been used in various sports, such as swimming [4, 7, 17], running [9, 33], cycling [16], resistance training [34, 35], water polo [6, 18] and repeated sprint ability [6]. The dosages vary among the studies available in the literature, with fixed quantities (1.6 g up to 6.4 g∙day^− 1^) and body mass (0.3 g∙kg^− 1^) [9, 33, 34]. Therefore, β-alanine’s function is to avoid intramuscular pH variations and is commonly combined with others ergogenic substances (buffer or stimulation) such as creatine, [33] or sodium bicarbonate (SB) [7, 33].

De Salles Painelli et al. (2013) investigated the effects of two supplementation strategies (β-alanine alone and in combination with SB) on 100-m and 200-m swimming performance. In β-alanine alone, sixteen swimmers received 3.2 g·day^− 1^ for 1 week and 6.4 g·day^− 1^ for 4 weeks and combining with SB (0.3 g∙kg^− 1^) on the last day of tests. Their main findings were that β-alanine supplementation, β-alanine supplementation with SB addition to the effort and only SB addition to effort effectively similarly improved 200-m freestyle performance, and tended to improve 100-m performance [17].

The β-alanine supplementation dose and time of exposure on the study performed by Salles Painelli et al. (2013) were similar to our study; however, the authors found no significant differences in buffer capacity (fatigue index) or ergogenic aid (peak and mean force, impulse and performance) were found [17]. This can be attributed to the metabolic characteristics of 400-m effort, around 80% of total energy demand is from of the aerobic system [1, 36], in comparison with the findings by Figueiredo et al. (2011), who reported a metabolic contribution of 65.9% Aer, 13.6% AnLa, and 20.4% AnAl during 200-m performance.

Mero et al. (2013) investigated the effects of a six-week supplementation using only β-alanine (4.8 g·day^− 1^) or PLA and combined co-administration of SB or PLA before two 100-m maximum freestyle bouts. Although pH increase was observed in both groups with SB, performance improvement was only observed when compared with PLA alone, and no differences were found for blood lactate concentration, evidencing that β-alanine supplementation did not provide significant alterations and effects were recurrent from SB administration. Under similar conditions of supplementation (time and quantity), the authors reported no alterations in performance, corroborating the present study. However, it should be noted that no changes in blood lactate concentration can be linked to the effort’s duration [1]. In addition, the non-parametric statistics used by Mero et al. (2013) may have limited the results of their analysis.

Nevertheless, a recent review [19] showed that β-alanine supplementation could have some influence in exercises lasting from 30s to 10 min. The 400-m performance in the present study was around 290 s, slightly longer than 4 min, and two studies [7, 17] on 100-m performance, where time was slightly shorter than 60 s, reported no significant effects of β-alanine supplementation. However, during a 200-m maximum effort, De Salles Painelli et al. (2013) observed significant improvement.

Chung et al. (2012) administrated 4.8 g·day^− 1^ of β-alanine for 4 weeks, following 6 weeks of 3.2 g·day^− 1^ or placebo, totalizing 10 weeks. Performances were assessed in important competitions, National Championships and international or national selection meet, and supplementation was conducted between them. The authors concluded that 10 weeks of β-alanine supplementation was not capable of improving physiological or performance benefits in a non-laboratory controlled, real-world competition, in elite/sub-elite swimmers.

Finally, although several studies have demonstrated that β-alanine supplementation can improve high intensity exercise performance [3, 4, 17], De Salles Painelli et al. (2013) stated that fewer studies have examined the effects on competitive performance. In the attempt to fill this gap, the present study demonstrated that β-alanine was not effective in increasing performance in 400-m. Nevertheless, new investigations are needed to determine which sports could benefit from the ergogenic effect of β-alanine (performance and training quality in speed and acidosis tolerance sets, 800-m running, team and combat sports).

The present study has some limitations, only three studies had their focus on swimming performance analysis, and none has quantified the metabolic contribution on β-alanine supplementation to make comparisons with the same method of quantification and modality. Therefore, this is the first study focused on shortening this gap in the literature. Even from the evidence of Stegen et al. (2013) the lack of intramuscular carnosine content measurement can be considered a limitation. Despite not measuring intramuscular carnosine, Harris et al. (2006) evidenced that the dose used in the present study seems sufficient to increase intramuscular content of carnosine even in a short period of supplementation. The chosen dose of β-alanine caused paresthesia on three athletes, showing which group was PLA and which was β-alanine. Finally, there were no nutritional control during supplementation, as feeding may influence the amount of carnosine originated by β-alanine supplementation.

Despite the methodologic constraints inherent in studies involving competitive athletes (e.g., reduced sample size, competition schedule, inability to perform invasive methodologies), they are important to substantiate the efficiency of nutritional aids over performance and metabolic contribution in sports.

## Conclusion

Although metabolic, neuromuscular and energy provision parameters changes, no effect from supplementation was found, evidencing that all the alterations found were the result of exposure to training. Thus, it can be concluded that β-alanine supplementation doesn’t changed metabolic contribution and it was ineffective in improving performance during 400-m freestyle.

## Data Availability

The datasets used and analyzed during the current study are available from the corresponding author on reasonable request.

## Abbreviations

[La^-^]: Blood lactate concentrations
H^+^: Hydrogen ions
β-alanine: Beta-alanine
Ca^++^: Calcium
TAn: Total anaerobic contribution
30ST: 30-s all-out tethered swimming effort
PLA: Placebo
EPOC_FAST_: Fast component of excess post-exercise oxygen consumption
AnAl: Anaerobic alactic contribution
[La^-^]_rest_: Blood lactate at rest
[La^-^]_PEAK_: Peak of blood lactate concentration
∆[La^-^]: Difference between [La-]_rest_ and [La-]_PEAK_
AnLa: Anaerobic lactic contribution
Aer: Aerobic contribution
PF: Peak force
MF: Mean force
FI: Fatigue index
IM: Impulse
Z1: Low intensity training
Z2: Medium intensity training
Z3: High intensity training

## References

[1] Campos EZ, Kalva-Filho CA, Gobbi RB, Barbieri RA, Almeida NP (2017). Anaerobic contribution determined in swimming distances: relation with performance. Front Physiol.

[2] Kalva Filho CA, Zagatto AM, Castanho Araújo MI, Pereira Santiago PR (2014). Ramos da Silva AS, Gobatto CA, et al. relationship between aerobic and anaerobic parameters from 3-min all-out tethered swimming and 400-m maximal front crawl effort. J Strength Cond Res.

[3] Hobson RM, Saunders B, Ball G, Harris RC, Sale C (2012). Effects of β-alanine supplementation on exercise performance: a meta-analysis. Amino Acids.

[4] Chung W, Shaw G, Anderson ME, Pyne DB, Saunders PU, Bishop DJ (2012). Effect of 10 week beta-alanine supplementation on competition and training performance in elite swimmers. Nutrients..

[5] de Andrade Kratz C, De Salles Painelli V, de Andrade Nemezio KM, Da Silva RP, Franchini E, Zagatto AM, et al. Beta-alanine supplementation enhances judo-related performance in highly-trained athletes. J Sci Med Sport. 2016; in press:10–5. 10.1016/j.jsams.2016.08.014 27601217

[6] Brisola GMP, Artioli GG, Papoti M, Zagatto AM (2016). Effects of four weeks of β-alanine supplementation on repeated sprint ability in water polo players. PLoS One.

[7] Mero AA, Hirvonen P, Saarela J, Hulmi JJ, Hoffman JR, Stout JR (2013). Effect of sodium bicarbonate and beta-alanine supplementation on maximal sprint swimming. J Int Soc Sports Nutr.

[8] Baguet A, Koppo K, Pottier A, Derave W (2010). β-Alanine supplementation reduces acidosis but not oxygen uptake response during high-intensity cycling exercise. Eur J Appl Physiol.

[9] Derave W, Ozdemir MS, Harris RC, Pottier A, Reyngoudt H, Koppo K (2007). Beta-alanine supplementation augments muscle carnosine content and attenuates fatigue during repeated isokinetic contraction bouts in trained sprinters. J Appl Physiol.

[10] Hill CA, Harris RC, Kim HJ, Harris BD, Sale C, Boobis LH (2007). Influence of beta-alanine supplementation on skeletal muscle carnosine concentrations and high intensity cycling capacity. Amino Acids.

[11] Culbertson JY, Kreider RB, Greenwood M, Cooke M (2010). Effects of Beta-alanine on muscle carnosine and exercise performance: a review of the current literature. Nutrients..

[12] Sale C, Saunders B, Harris RC (2010). Effect of beta-alanine supplementation on muscle carnosine concentrations and exercise performance. Amino Acids.

[13] Artioli GG, Gualano B, Smith A, Stout J, Lancha AH (2010). Role of β-alanine supplementation on muscle carnosine and exercise performance. Med Sci Sports Exerc.

[14] Juel C (2007). Changes in interstitial K+ and pH during exercise: implications for blood flow regulation. Appl Physiol Nutr Metab.

[15] Harris RC, Tallon MJ, Dunnett M, Boobis L, Coakley J, Kim HJ (2006). The absorption of orally supplied beta-alanine and its effect on muscle carnosine synthesis in human vastus lateralis. Amino Acids.

[16] Bellinger PM, Minahan CL (2016). Metabolic consequences of beta-alanine supplementation during exhaustive supramaximal cycling and 4000-m time-trial performance. Appl Physiol Nutr Metab.

[17] De Salles PV, Roschel H, de Jesus F, Sale C, Harris RC, Solis MY (2013). The ergogenic effect of beta-alanine combined with sodium bicarbonate on high-intensity swimming performance. Appl Physiol Nutr Metab.

[18] Claus GM, Redkva PE, Brisola GMP, Miyagi WE, Moura AM. β-Alanine Supplementation Improves Throwing Velocities in Repeated Sprint Ability and 200-m Swimming Performance in Young Water Polo Players. Pediatr Exerc Sci. 2017;29(2):203–212. 10.1123/pes.2016-0176 28121265

[19] Saunders B, Elliott-Sale K, Artioli GG, Swinton PA, Dolan E, Roschel H, et al. β -alanine supplementation to improve exercise capacity and performance: a systematic review and meta-analysis. Br J Sports Med. 2016;51(8):658–669. 10.1136/bjsports-2016-096396 27797728

[20] Brisola GMP, Redkva PE, Pessoa Filho DM, Papoti M, Zagatto AM (2018). Effects of 4 weeks of β-alanine supplementation on aerobic fitness in water polo players. PLoS One.

[21] Brisola GMP, Zagatto AM. Ergogenic Effects of β-Alanine Supplementation on Different Sports Modalities: Strong Evidence or Only Incipient Findings? J Strength Cond Res. 2018;1 Available from: http://insights.ovid.com/crossref?an=00124278-900000000-95053. 10.1519/JSC.0000000000002925 30431532

[22] da Silva RP, de Oliveira LF, Saunders B, de Andrade Kratz C, de Salles Painelli V, da Eira Silva V, et al. Effects of β-alanine and sodium bicarbonate supplementation on the estimated energy system contribution during high-intensity intermittent exercise. Amino Acids. 2018;51(1):83–96. 10.1007/s00726-018-2643-2 30182286

[23] Montpetit RR, Léger LA, Lavoie JM, Cazorla G (1981). VO2 peak during free swimming using the backward extrapolation of the O2 recovery curve. Eur J Appl Physiol Occup Physiol.

[24] Kalva-Filho CA, Araújo MYC, Silva A, Gobatto CA, Zagatto AM, Gobbi RB, et al. Determination of VO2-Intensity relationship and MAOD in tethered swimming. Int J Sports Med. 2016;37(9):687–93. 10.1055/s-0035-1559696 27176891

[25] Campos EZ, Kalva-Filho CA, Loures JP. Comparison between peak oxygen consumption and its associated speed determined through an incremental test and a 400-m effort: implication for swimming. Sci Sports. 2017;32(1):e37-e41.

[26] Zagatto A, Redkva P, Loures J, Kalva Filho C, Franco V, Kaminagakura E (2011). Anaerobic contribution during maximal anaerobic running test: correlation with maximal accumulated oxygen deficit. Scand J Med Sci Sports.

[27] Bertuzzi RCM, Franchini E, Ugrinowitsch C, Kokubun E, Lima-Silva AE, Pires FO (2010). Predicting MAOD using only a supramaximal exhaustive test. Int J Sports Med.

[28] Di Prampero PE, Ferretti G (1999). The energetics of anaerobic muscle metabolism: a reappraisal of older and recent concepts. Respir Physiol.

[29] Figueiredo P, Zamparo P, Sousa A, Vilas-Boas JP, Fernandes RJ (2011). An energy balance of the 200 m front crawl race. Eur J Appl Physiol.

[30] Seiler KS, Kjerland GØ (2006). Quantifying training intensity distribution in elite endurance athletes: is there evidence for an “optimal” distribution?. Scand J Med Sci Sports.

[31] Lee MD, Wagenmakers EJ (2013). Bayesian cognitive modeling: a practical course. Bayesian Cognitive Modeling: A Practical Course.

[32] Damasceno MV, Pasqua LA, Lima-Silva AE, Bertuzzi R (2015). Energy system contribution in a maximal incremental test: correlations with pacing and overall performance in a 10-km running trial. Braz J Med Biol Res.

[33] Stout JR, Cramer JT, Mielke M, O’Kroy J, Torok DJ, Zoeller RF (2006). Effects of Twenty-eight days of beta-alanine and creatine monoydrate supplementation on the physical working capacity at neuromuscular fatigue threshold. J Strength Cond Res.

[34] Hoffman JR, Ratamess N, Ross R, Kang J, Magrelli J, Neese K (2008). β-Alanine and the hormonal response to exercise. Int J Sports Med.

[35] Rodriguez F, Mader A. Energy metabolism during 400 and 100-m crawl swimming: computer simulation based on free swimming measurement. Biomech Med Swim VIII. 2003;9(1):373–78.

[36] Stegen S, Blancquaert L, Everaert I, Bex T, Taes Y, Calders P (2013). Meal and beta-alanine coingestion enhances muscle carnosine loading. Med Sci Sports Exerc.

