# Correction to: Beta alanine supplementation effects on metabolic contribution and swimming performance

**DOI:** 10.1186/s12970-020-00380-7

**Published:** 2020-10-08

**Authors:** Matheus Silva Norberto, Ricardo Augusto Barbieri, Danilo Rodrigues Bertucci, Ronaldo Bucken Gobbi, Eduardo Zapaterra Campos, Alessandrou Moura Zagatto, Ellen Cristini De Freitas, Marcelo Papoti

**Affiliations:** 1 University of São Paulo, Medicine University of Ribeirão Preto (FMRP-USP), Ribeirão Preto, São Paulo, Brazil; 2 University of São Paulo, School of Physical Education and sport of Ribeirão Preto (EEFERP-USP), Ribeirão Preto, São Paulo, Brazil; 3 Estácio University, Ribeirão Preto, São Paulo, Brazil; 4 Department of Physical Education, State São Paulo University, (UNESP), Rio Claro, São Paulo, Brazil; 5 Department of Physical Education, Federal University of Pernambuco, (UFPE), Recife, Pernambuco Brazil; 6 Department of Physical Education, State São Paulo University, (UNESP), Bauru, São Paulo, Brazil

**Correction to: J Int Soc Sports Nutr 17, 40 (2020)**

**https://doi.org/10.1186/s12970-020-00365-6**

The original article [1] contains a typo in co-author, Alessandrou Moura Zagatto’s name. The correct presentation can be viewed in this Correction article.
